# Facial Bone Defects Associated with Lateral Facial Clefts Tessier Type 6, 7 and 8 in Syndromic Neurocristopathies: A Detailed Micro-CT Analysis on Historical Museum Specimens

**DOI:** 10.3390/biology14070872

**Published:** 2025-07-17

**Authors:** Jana Behunova, Helga Rehder, Anton Dobsak, Susanne G. Kircher, Lucas L. Boer, Andreas A. Mueller, Janina M. Patsch, Eduard Winter, Roelof-Jan Oostra, Eva Piehslinger, Karoline M. Reich

**Affiliations:** 1 Institute of Medical Genetics, Medical University of Vienna, 1090 Vienna, Austria; jana.behunova@meduniwien.ac.at (J.B.); helga.rehder@alumni-club.meduniwien.ac.at (H.R.); susanne.kircher@alumni-club.meduniwien.ac.at (S.G.K.); 2 Karl Donath Laboratory, University Clinic of Dentistry, Medical University of Vienna, 1090 Vienna, Austria; anton.dobsak@meduniwien.ac.at; 3 Department of Imaging, Section Anatomy and Museum for Anatomy and Pathology, Radboud University, Medical Center, 6525 Nijmegen, The Netherlands; lucas.boer@radboudumc.nl; 4 Department of Oral and Craniomaxillofacial Surgery, University Hospital Basel and University Children’s Hospital Basel, 4056 Basel, Switzerland; andreas.mueller@usb.ch; 5 Facial and Cranial Anomalies Research Group, Department of Clinical Research, University of Basel, 4001 Basel, Switzerland; 6 Department of Biomedical Imaging and Image-Guided Therapy, Division of General and Pediatric Radiology, Medical University of Vienna, 1090 Vienna, Austria; janina.patsch@meduniwien.ac.at; 7 Pathological-Anatomical Collection in the Narrenturm, Natural History Museum Vienna, 1090 Vienna, Austria; eduard.winter@nhm-wien.ac.at; 8 Department of Medical Biology Section Clinical Anatomy and Embryology, Amsterdam University Medical Center, Academic Medical Center, University of Amsterdam, 1098 Amsterdam, The Netherlands; r.j.oostra@amsterdamumc.nl; 9 Department of Prosthodontics, University Clinic of Dentistry, Medical University of Vienna, 1090 Vienna, Austria; eva.piehslinger@meduniwien.ac.at

**Keywords:** lateral facial clefts, congenital facial bone defects, maxillomandibular complex, neural crest cells, Treacher Collins syndrome, acrofacial dysostosis type Rodriguez, tetra-amelia, agnathia, fetal skulls, Narrenturm Museum Vienna

## Abstract

Lateral facial clefts are rare and often part of more complex syndromic neurocristopathies. According to Tessier’s classification, they correspond to facial cleft numbers 6, 7 and 8. Using micro-computer tomography (micro-CT), we analyzed their underlying bone defects (resolution 50 and 55 µm/voxel) in the context of the known syndrome-specific genetic background. Lateral facial clefts were diagnosed in three severely affected museum specimens representing mandibulofacial dysostosis type Treacher Collins syndrome (TCS), acrofacial dysostosis syndrome of Rodriguez (AFD-Rod) and tetra-amelia syndrome (TETAMS). The TCS specimen mainly showed an absence of the zygomatic bones and most of the lateral maxilla. The AFD-Rod specimen showed an extensive defect of the lateral maxilla, zygomatic bones, and mandible. The TETAMS specimen showed almost isolated agnathia. Possible relationships are discussed between the diverse facial bone defects due to apoptosis of neural crest-derived cells, known to be associated with ribosomopathies and spliceosomopathies, such as TCS and AFD-Rod, and the more targeted bone defects due to genetic variants known to cause TETAMS.

## Simple Summary

Lateral facial clefts (Tessier types 6–8) are rare in contrast to the common paramedian cleft lip and palate. They can occur as single malformations but are often part of malformation syndromes and may affect soft tissue structures and underlying bones. Although knowledge of the anatomical changes involved is crucial for understanding disease mechanisms and improving treatment options, detailed morphological analyses of lateral facial clefts are lacking. This study aimed to analyze the facial bone defects in three severely affected fetal museum specimens representing Treacher Collins syndrome (TCS), acrofacial dysostosis syndrome of Rodriguez (AFD-Rod) and tetra-amelia syndrome (TETAMS). Using micro-computer tomography, it was possible to identify distinct alterations in facial bone size, shape, fusions, and disintegration reflecting different gene defects critical in facial development. Possible relationships are discussed between the diverse facial bone defects due to oxidative stress-induced death of neural crest-derived cells, known to be associated with TCS and AFD-Rod, and the more targeted bone defects due to genetic variants known to cause TETAMS. Understanding the morphological manifestations of lateral facial clefts in relation to their genetic background provides valuable insights for both developmental researchers as well as for clinicians diagnosing and classifying congenital clefts and performing surgical reconstructions.

## 1. Introduction

Lateral facial clefts are a heterogeneous group of rare developmental anomalies of the skeletal and soft tissues of the face, distinct from the more common cleft lip and palate group and the rarer median and paramedian facial clefts [1]. They may be accompanied by maldevelopment of the eyes, ears, nose and/or other local structures. The overall incidence of atypical facial clefts, including lateral clefts, is estimated to be between 1.4 and 4.9 per 100,000 live births [2].

Lateral facial clefts can occur as an isolated malformation, such as macrostomia, or in association with various systemic anomalies [3,4]. They may result from teratogenic disruptions of initially normal embryonic development, including mechanical factors, or may be genetic or a feature of a monogenic syndrome4 such as Treacher Collins syndrome (TCS). TCS is characterized by mandibulofacial dysostosis (MFD) with zygomytic and mandibular hypoplasia, downslanting palpebral fissures, lower eyelid colobomas, microtia, and conductive hearing loss, as well as macrostomia [5]. It is also conceivable that lateral facial clefts occur in other syndromes characterized by severe MFD, such as the acrofacial dysostosis (AFD) syndromes. In AFD, craniofacial anomalies are associated with limb anomalies or even phocomelia, as seen in AFD type Rodriguez [6]. However, lateral facial clefts are an unexpected finding in syndromes in which extrafacial features predominate. One such example is tetra-amelia syndrome (TETAMS), which is occasionally associated with pulmonary aplasia and diaphragmatic hernia [7].

**Figure 1:**
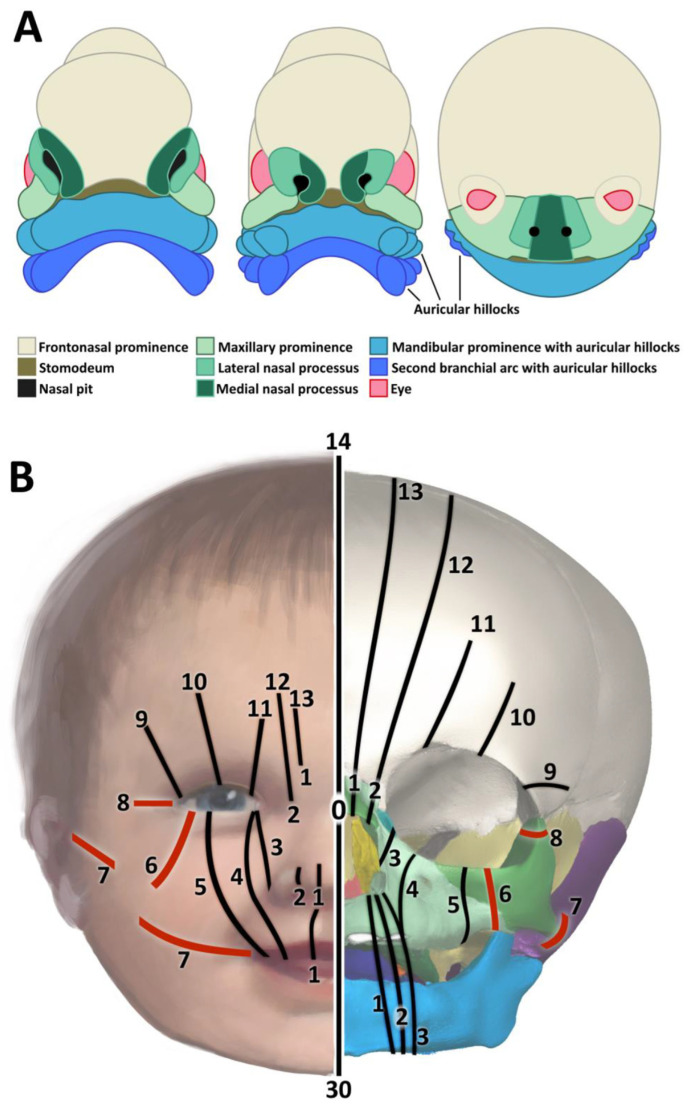
Facial development and Tessier classification of facial clefts. (**A**) Facial development. (**B**) Tessier classification of facial clefts. TC-0, 14, 30—median, TC-1, 2, 13—paramedian (including cleft-lip-palate), TC-3—oculo-nasal (paranasal), TC-4—oculo-facial I. TC-5—oculo-facial II., TC-6,7,8—lateral facial clefts: TC-6—maxillary–zygomatic, from infraorbital fissure, along the maxillary–zygomatic suture (bones) and from lateral lower eyelid downwards and slightly laterally (soft tissue), TC-7—temporo-zygomatic (transversal), from mouth corner to ear pit, TC-8—fronto-zygomatic, along the fronto-zygomatic suture (bone) with cleft of lateral canthus (soft tissue), TC-9 –12—cranial counterparts of 5-2.

The most widely used morphoanatomical classification of facial clefts is that of Tessier (Figure 1B) [8]. Tessier categorized facial clefts by location, assigning them numbers from 0 to 14. Clefts 0 to 7 are primarily facial, while clefts 8 to 14 are cranial. Lateral facial clefts correspond to Tessier numbers 6, 7 and 8. Tessier no. 6 is a maxillary–zygomatic oblique cleft. Bony defects include a short posterior maxilla, hypoplastic or absent zygomatic bones, a high-arched palate, and sometimes choanal atresia. Soft tissue defects usually present as discrete, scar-like lines rather than complete tissue separation. These lines extend from the upper lip to the lateral third of the lower eyelid, causing colobomas and downslanting palpebral fissures. Tessier no. 7, also known as temporo-zygomatic or transverse cleft, is often associated with absence or hypoplasia of the zygomatic bones and arches, deformities of the mandibular rami, condyles and coronoid processes, vertically short maxilla, and hypoplastic alveoli. Soft tissue defects may include temporal muscle hypo-or aplasia, preauricular tags, and macrostomia with extension of the corners of the mouth into the cleft. In Tessier no. 8, which corresponds to a fronto-zygomatic cleft, the hypo-/aplasia of the zygomatic bones results in a defect of the lateral orbital rims that are formed only by the greater sphenoid wings. Sometimes a subtle sunken line extending from the lateral canthus to the temporal region, or a soft tissue cleft of the lateral canthus, is present [8].

To date, most studies on lateral facial clefts have focused on surgical repair techniques [3,9,10], and although knowledge of the anatomical changes involved is crucial to understanding disease mechanisms, morphological analyses of lateral facial clefts are mostly lacking [11]. The Pathological Anatomical Collection in the Narrenturm (Fools Tower) in Vienna is one of the worldwide largest collections of pathological anatomy research and is a unique resource for the analysis of rare developmental disorders. These museum specimens have the advantage of allowing us to apply high-intensity, high-resolution imaging techniques such as micro-computer tomography (micro-CT), revealing valuable 3-dimensional morphological details. The construction of the Narrenturm building, one of the first institutions globally dedicated to housing and treating the mentally ill, was commissioned in 1778 by Emperor Joseph II. Since 1971, it has housed the Pathological–Anatomical Museum, with nearly 50,000 specimens [12,13].

This study aims to provide new morphological insights into the severe manifestations of lateral facial clefts not only by studying the dysmorphological aspects of particularly severely affected museum specimens but also by uncovering the underlying bone defects using micro-CT. A better understanding of how lateral facial clefts affect facial skeletal structures, in the context of their known syndrome-specific genetic background, is important for researchers focusing on developmental aspects, as well as for clinicians, radiologists and geneticists involved in the prenatal and postnatal diagnosis of congenital anomalies, their syndromic assignment, and the performance of surgical reconstructions.

### Facial Development

Craniofacial development involves a cascade of highly coordinated processes that rely on the seamless spatiotemporal interaction of cranial ectomesenchymal neural crest cells, surface ectoderm, pharyngeal endoderm, paraxial and lateral plate mesoderm, and sensory placodes [14]. One of the earliest morphological signs of facial development is the appearance of a central primitive mouth pit (stomodeum) delineated cranially by the frontonasal prominence and laterally by the first pair of pharyngeal arches that are formed within the primitive pharyngeal wall and give rise to paired maxillary and mandibular prominences (Figure 1A). These prominences, derived from migrating, mesenchymally transformed neural crest cells, are established within developmental week (DW) 4 and represent key structures for facial development [15]. Three paired placodes, the nasal, lenticular and otic placodes, arise almost simultaneously at the sides of the head region. The frontonasal prominence proliferates around the nasal placodes forming the paired medial and lateral nasal processes. The medial nasal processes fuse to form the intermaxillary structures comprising the midline of the nose, philtrum, premaxilla with four incisors, and primary palate, the earliest separation of oral and nasal cavity. The maxillary prominences give rise to the lateral parts of the upper lip, midfacial bones, maxilla, and to the secondary palate. Recent evidence suggests that the mammalian premaxilla is composed of both frontonasal and maxillary neural crest cells [16].

In DW 6 and 7, the maxillary prominences grow medially, approaching the nasal processes to complete the formation of the maxilla and palate. The fusion of the primary and secondary palate completes the separation of the oronasal cavity into oral and nasal parts. Failed fusion of the maxillary prominences with medial nasal processes and of primary with secondary palate results in a common ‘cleft lip and palate’, Tessier no. 2 (Figure 1B). The lateral nasal processes remain temporarily separated from the maxillary prominences forming the nasolacrimal grooves that later become the nasolacrimal ducts. Failure of duct closure and defective outgrowth of the lateral nasal processes and maxillary prominences result in orbito-maxillary clefts, Tessier no. 3 and 4 (Figure 1B). The mandibular prominences grow medially and fuse to form the lower jaw, lower lip and chin. Failed fusion results in a rare median mandibular cleft with cleft lower lip. The lateral maxillae, the zygomatic bones, the greater wings of the sphenoid, and the squamosal part of the temporal bones arise from neural crest cells of the maxillary prominences [17]. The temporomandibular joints develop between the condylar processes of the mandible and the glenoid blastema of the temporal bones. The superficial parts of the maxillary and mandibular prominences fuse laterally in the DW 5–8 to form the buccal muscles. Failed fusion results in lateral cleft Tessier no. 7, which clinically imposes as macrostomia. Tessier no. 7 is often associated with impaired fusion of the maxillary ossification centers, and by this also with Tessier clefts no. 6 and 8 (Figure 1B). It is worth noting, however, that the localization of clefts typically occurs along the epithelial fusion lines between the merging facial prominences/processes and does not necessarily correspond to the sutures of the (facial) bones [18].

Ear development begins in DW 3 with the invagination of the ectodermal otic placodes as the otic pits, which, after closure, form the otocysts. They give rise to the membranous labyrinth of the inner ear with the cochlea, vestibule and three semicircular canals. The surrounding mesenchyme begins to chondrify in DW 9 and ossifies between DW 16 and 23 to form the bony labyrinths and the petrous and mastoid parts of the temporal bones [15]. The middle ear cavities originate from the endoderm of the first pharyngeal pouches. The ossicles, malleus and incus arise from the posterior end of the Meckel’s cartilage within the mandibular prominences of the first pharyngeal arches, while the stapes arises from the second pharyngeal arch. The neural crest-derived mesenchyme of the first and second pharyngeal arches also forms six auricular hillocks around the ear pits. They fuse, chondrify, and move upward from their low cervical position. The auditory canals are formed from the ectodermal first pharyngeal clefts, opposite the first pharyngeal pouches, with the mesenchyme between them forming the tympanic membranes [19,20].

Tooth development begins in DW 5 as a proliferation of epithelial cells in the maxillary and mandibular ridges (odontogenic epithelium), forming dental and vestibular lamina. In the dental lamina, tooth germs develop because of a complex interaction between ectodermal and neural crest cells. The tooth germs proliferate and differentiate as they pass through the characteristic bud, cap and bell stages. After DW 14, the odontoblasts and ameloblasts begin secreting dentin and enamel, respectively, to form the tooth crowns. Dental roots are formed by the apical apposition of root dentin and cementum after crown formation is complete. Mesenchymal cells of the dental sacs form the periodontal ligaments connect the erupting deciduous teeth to the alveolar bone that is formed via intramembranous ossification. The main part of the maxillary and mandibular bodies develops in DW 6–8, and the formation of their alveolar parts is linked to the eruption of teeth [21,22].

## 2. Materials and Methods

The study was conducted according to the guidelines of the Declaration of Helsinki and approved by the Ethics Committee of the Medical University of Vienna (Approval No: 1312/2012). Three formalin-preserved human specimens with lateral facial clefts from the Pathological–Anatomical Collection of the Narrenturm Museum in Vienna were examined in this study. Specimen 1 was sent to the Narrenturm in 1947 with the diagnosis ‘Newborn at 40 weeks gestation with anotia bilateralis, monstrositates multiplices’. No autopsy was performed. Specimen 2 was transferred from the Natural History Museum in Salzburg, Austria, in 2005 with the diagnosis ‘phocomelia’, but no year of birth or gestational age was given. An autopsy was performed, but there is no autopsy report. Specimen 3 came from the Pathology collection of the General Hospital in Vienna in 1976 with the diagnosis ‘amelos’. Again, no year of birth or gestational or postnatal age was given. No autopsy was performed.

After physical and X-ray examination of the individuals, the findings were documented photographically. Whole-body CT scans were performed in specimens 2 and 3 using a Siemens Naeotom Alpha (Photon-Counting Detector-CT, Siemens Healthineers, Forchheim, Germany). For a detailed assessment of the facial bones, the skulls of all three specimens were scanned by use of a micro-CT scanner (VISCOM X8060 NDT, Viscom AG, Hannover, Germany), with an isotropic resolution between 50 and 55 µm/pxl at 130 kV, 380 µA, with 1400 projections per 180°, and an exposure time of 1400 ms. A 0.75 mm copper filter was used to reduce radiation hardening. The scan data sets were reconstructed using the scanner’s reconstruction software and then rendered and further processed using the 3D visualization software AMIRA Version 2020.2 (Thermo Fisher Scientific, Waltham, MA, USA). The dynamic range was manually optimized by a trained expert, with consideration of beam hardening artefacts present in the scans.

By rotating the 3D reconstructed skulls, reducing the opacity of the rendered surface, and placing virtual planes through the regions of interest, we were able to visualize the cleft region in any desired orientation and gain insight into the internal osseous structure of the defect. The individual skull bones were segmented and labelled with different colors to facilitate their identification. Age at death was estimated using the London Atlas method, based on dental development and eruption status of the teeth [23]. Micro-CT scans of three specimens of comparable age at death, without facial anomalies from an osteological collection of infant skulls (V. Terzer, early 19th century, housed in the Karl Donath Laboratory, University Clinic of Dentistry, Medical University of Vienna), were used as reference. Reference individual 1 died at 4 days of age, and the cause of death was not documented. Reference individual 2 died during a Caesarean delivery (age at death: 7 months in utero). Reference individual 3 died at the age of 1 month, and the cause of death was not documented.

A qualitative description of the malformations affecting the midfacial bones, jaws, tooth germs, cranial base and the middle and inner ears was performed and compared with the age-matched reference individuals. The bone analysis was performed jointly by J.B., K.M.R., A.D., S.G.K., and H.R. by mutual agreement. In the very rare cases where the identification and delineation of the anatomical structures was not clear, joint discussions were held until consensus was reached. Morphometric standard parameters according to Bräuer (1988) [24], and other relevant anatomical structures such as ear canals, were interactively measured using the AMIRA software, Version 2020.2 (Thermo Fisher Scientific, Waltham, MA, USA). (Please also see Supplementary Table S1).

## 3. Results

The morphological features observed in our three specimens are summarized in Table 1. Morphometric measurements of the maxilla, hard palate and mandible are provided in Supplementary Table S1. Three-dimensional animations illustrating the micro-CT reconstructions of specimen 1, 2 and 3 are included as a Supplementary File (Supplementary Videos S1, S2 and S3).

### 3.1. Specimen 1 (MN.18.560/1836)

**Figure 2:**
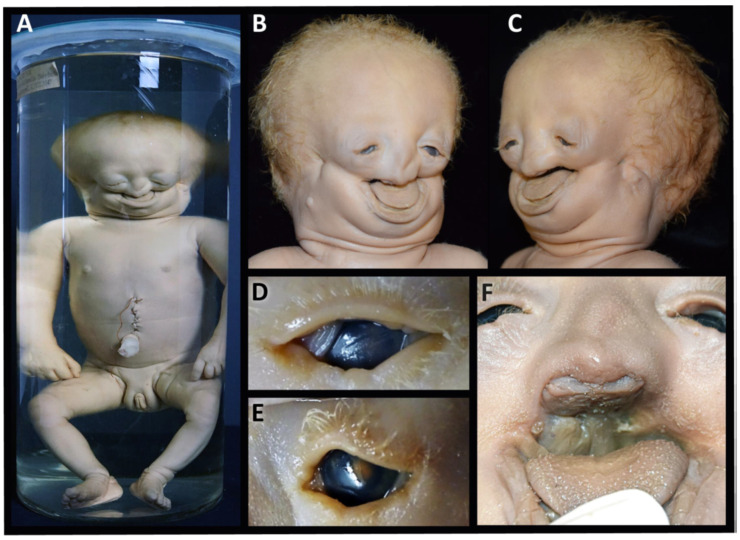
Specimen 1: Clinical diagnosis of Treacher Collins syndrome. Female newborn showing severe mandibulofacial dysostosis without other external body abnormalities (**A**). Facial features with a broad forehead, severely hypoplastic midface, microretrogenia, lacking auricles, residual skin tag below the ear pits, hypertelorism, downslanting narrow palpebral fissures (**B**,**C**); eyelid colobomas (**D**,**E**); flat nose with absent nasal orifices, philtrum and upper lip and macroglossia (**F**). The transverse orofacial clefts (TC-7) present as macrostomia, merging bilaterally into a deep linear groove directed towards the ear pits (**B**,**C**). (**A**) is reproduced from Boer et al. (2023) [12], with permission from Wiley, licensed by CC BY.

Clinical features: The specimen represents a 40 gestational-week-old, small for date, female newborn with a crown–rump length (CRL) of 29.5 cm, a crown–heel length (CHL) of 43.5 cm and a head circumference (HC) of 31 cm. It shows severe craniofacial anomalies, including a broad skull, a broad high forehead, bilateral anotia (microtia grade IV) [25,26], hypertelorism, downslanting narrow palpebral fissures with lower eyelid colobomas, a hypoplastic midface and a flat almost absent nose. The nasal orifices, philtrum and lips are completely absent, but there are bilateral nasal protuberances reminiscent to nasal wings (Figure 2A–C,F). The lower edge of the nose completely covers the premaxilla. There are bilateral transversal oral clefts directed towards the ear pits and merging halfway into a linear groove. There is also microretrogenia. The auricles are absent, with a residual skin tag 17 and 12 mm below the right and left atretic auditory canal. The trunk and the limbs are of normal structure (Figure 2A). The facial features are consistent with a severe form of Treacher Collins syndrome (OMIM # 154500, # 613717, # 248390, # 618939) associated with lateral facial clefts of Tessier no. 6, 7 and 8 [8].

**Figure 3:**
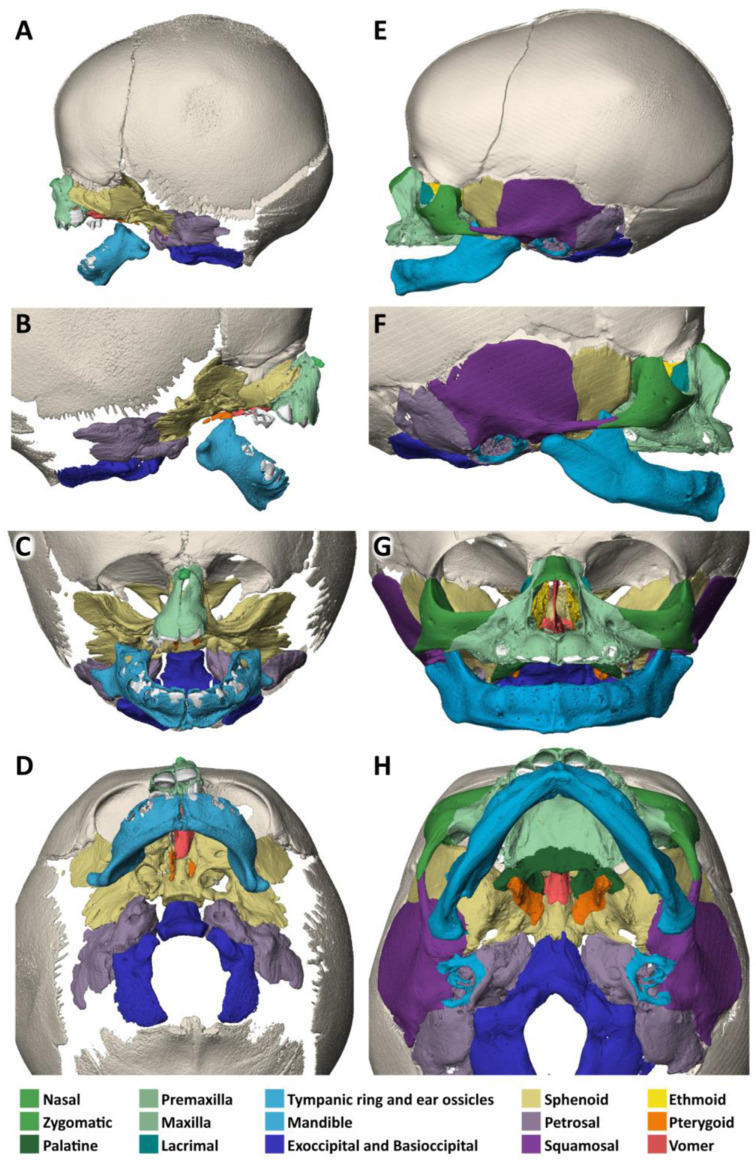
Specimen 1 and reference: Micro-CT-based 3D reconstruction of the skulls. Osseous defects in specimen 1, Treacher Collins syndrome (**A**–**D**). Reference 1, unaffected 4-day-old individual; Terzer collection (**E**–**H**). Lateral overview from the left (**A**,**E**) and from the right (**B**,**F**). Frontal view (**C**,**G**). Inferior view of the skull base (**D**,**H**). Individual skull bones are color-coded to facilitate comparison (corresponding colors in text). Absence of the zygomatic bones and most of the lateral maxillae, deformed mandibular angles and condylar processes, irregularly developed sphenoid bones fused with parts of the ethmoid, lacrimal and squamosal bones and the maxillae in specimen 1.

**Figure 4:**
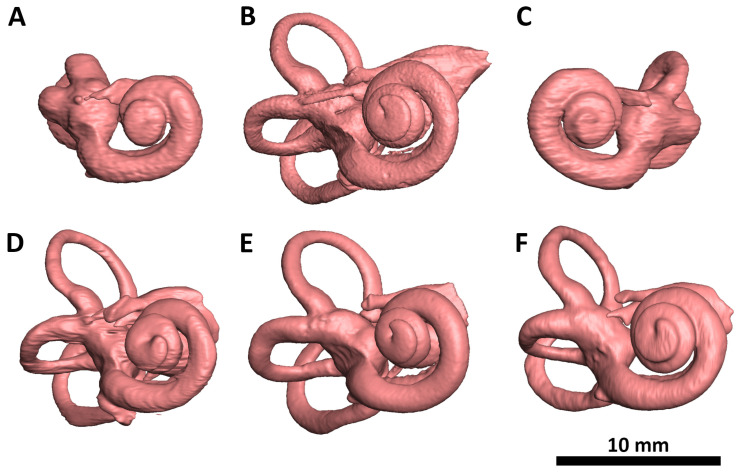
Micro-CT-based 3D reconstruction of middle and inner ears. (**A**,**C**) Specimen 1: Treacher Collins syndrome (right and left hypoplastic, clumped cochlea). (**B**) Reference individual 1 (left cochlea). (**D**) Specimen 2: Acrofacial dysostosis syndrome of Rodriguez (right normally developed cochlea). (**E**) Reference individual 2 (right cochlea). (**F**) Specimen 3: tetra-amelia syndrome (left normally-developed cochlea).

A micro-CT analysis of the head, when compared to a normal neonate, reveals a normal shape and size of the neurocranium (Figure 3A–H). The small accessory frontal bone patches above the premaxilla represent a normal variant. However, the facial bones are severely deformed:

- The nasal bone (dark green) is almost absent except for a very small cranial segment. A nasal septum is absent. The vomer (red) appears markedly broadened, frontally shortened and with hypoplastic dorsal wings. The two lamellae of the vomer are not fused cranially and an ossified attachment to a nasal septum is missing.
- The upper jaw (light green) is missing its lateral parts. A narrow median segment, representing the premaxilla, is displaced upwards. It encloses four deciduous incisors, which are developed according to the indicated age of approximately 40 weeks of gestation (±1 weeks) [23]. There are no canines. The cusps of the first right and left deciduous molar and a rudimentary second right deciduous molar are located in the soft tissue near the premaxillary bone and are not encased by a bony crypt. A median furrow of the premaxilla widens into a gap towards the rudimentary nasal bone, possibly representing the osseous nasal orifice and suggesting choanal stenosis. The premaxilla forms a primary palate, while the palatal shelves of the bilateral maxillary bones which should form the secondary palate are absent. The upper roof of the palate consists of a widened vomer and laterally of a bony conglomerate. There are no incisive, palatal or infraorbital foramina. There is a bifid uvula.
- The lower jaw (light blue) is shortened and shows a latero-caudal bend instead of a cranial bend. The ramus of the mandible and the coronoid process is hypoplastic and the condylar process is deformed and close to the angle of the mandible. A temporomandibular joint is bilaterally absent. All the teeth of the mandible are enclosed in osseous crypts and are within the normal range for age. The mandibular canals are recognizable as grooves. There is no midline fusion of the two halves of the mandible. The mental foramina are present, as are the mandibular canals and foramina located at the back of the deformed mandibular rami.
- The zygomatic bones are missing. The sphenoid, ethmoid, lacrimal, a rudimentary squamosal part of the temporal bones and possibly parts of the lateral maxillary bones appear to be irregularly fused into a single bony structure with no apparent boundaries (beige). The zygomatic and styloid processes are absent, as are the tympanic portions of the temporal bones (light violet). Foramina rotunda and ovalia of the skull base are not identifiable. The orbits are formed medially by the fused bony mass, and laterally and superiorly by the frontal bones. The inferior orbital ridges are lacking.
- The ears show absent auricles, with a residual skin tag. The length of auditory canals is 6 mm on the right and 11 mm on the left side. The tympanic membranes and middle ear cavities are lacking, except for a rudimentary intraosseous vesicular structure without ossicles, from which the air-filled Eustachian tubes branch off, although their course cannot be traced with certainty. Both inner ears show a hypoplastic and clumped cochlea without foramina but with obvious coils. The labyrinth has one thick semicircular canal on the right and two on the left. The remaining semicircular canals appear as rudimentary nodular projections (Figure 4A,C).

For the 3-dimensional animation of the micro-CT reconstruction of the skull, please refer to Supplementary Video S1.

### 3.2. Specimen 2 (MN.32.808/40)

**Figure 5:**
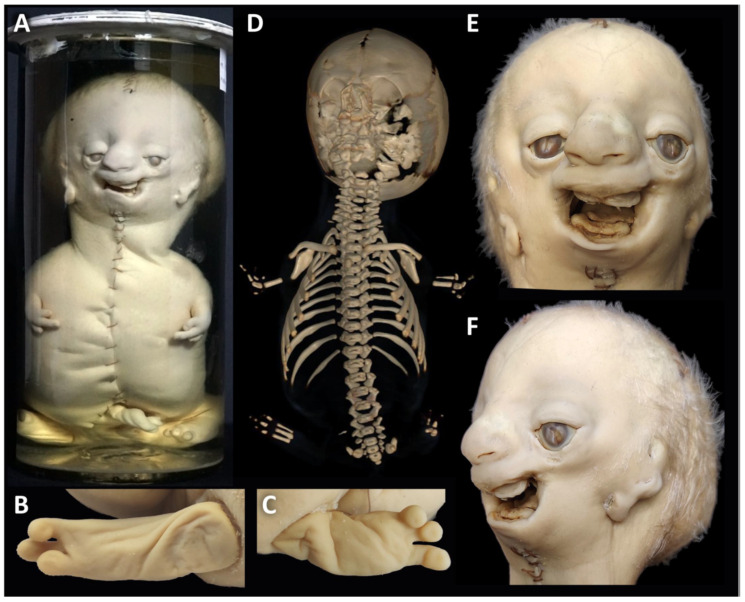
Specimen 2: Clinical diagnosis of acrofacial dysostosis syndrome of Rodriguez. Male fetus with severe mandibulofacial dysostosis and tetraphocomelia with three digits on the hands and feet (**A**–**C**). Whole-body CT with 3D reconstruction showing absent humeri, extremely shortened ulnae, absent or shortened radii, one long median and two short lateral digital rays bilaterally, hypoplastic scapulae, 11 ribs bilaterally, missing pelvic bones, femora, tibiae, fibulae and tarsals, and three digital rays on both feet (**D**). Macrostomia and furrows directed from the mouth corner to the temporal region, corresponding to transverse facial cleft (TC-7). Small cleft of lateral canthus (TC-8), hypertelorism, proptosis, large lower eyelid colobomas (TC-6), microretrogenia and low-set severely dysplastic ears (**E**,**F**). (**A**) Reproduced from Boer et al. (2023) [12] with permission from Wiley, licensed by CC BY.

Clinical features: The specimen corresponds to a male preterm neonate of 23 cm CRL and 29.4 cm HC with severe craniofacial anomalies and tetraphocomelia (Figure 5A). The head is dolichocephalic. The face shows a high forehead, hypertelorism, slightly downslanting palpebral fissures, lower and upper eyelid colobomas, most prominent in the middle third of the upper eyelids, proptosis, severe midfacial hypoplasia, short bulbous nose, smooth philtrum and pronounced microretrognathia. The low-set ears are severely dysplastic, corresponding to microtia grade III [25,26], with three lobule type auricles, lacking typical auricular structures like the helix, antihelix, tragus and antitragus. They are located 7 mm and 3 mm below the slit-like openings of the atretic auditory canals. The facial clefts do not distort the skin’s integrity, so they are not easily detectable. They run bilaterally from the corners of the mouth to the lateral canthi, slightly higher to the medial canthi, and to the ear tags on the right and to the ear pit on the left (Tessier no. 6, 7 and 8) (Figure 5A,E,F). The limbs are shortened to about 4 cm in length and have three digits with nails on each and with a soft tissue cleft between the middle and lateral fingers (Figure 5A,D). Both nipples are displaced downward along the milk line in about the middle of the trunk, the right one slightly lower. X-ray and a whole-body CT show dysmorphic clavicles and scapulae, absent humeri and extreme shortening of the ulnae and radii, with only a rudimentary right radius. There are only three metacarpals on each side, of which the central one is thickened and appears to consist of two fused metacarpals. There is a cleft formation on its lateral side. The thumbs are missing, the index and small fingers only show one short ossified phalanx. There is no bony pelvis except for two tiny bone fragments on either side of the 2nd sacral vertebra. Femora, tibiae and fibulae are missing. There are three metatarsals on the right side and two on the left (Figure 5B–D). The association of severe MFD including lateral facial clefts of Tessier no. 6, 7, and 8 with tetraphocomelia corresponds to the diagnosis of an acrofacial dysostosis syndrome of Rodriguez (OMIM %201170). AFD-Rod is an early lethal condition.

**Figure 6:**
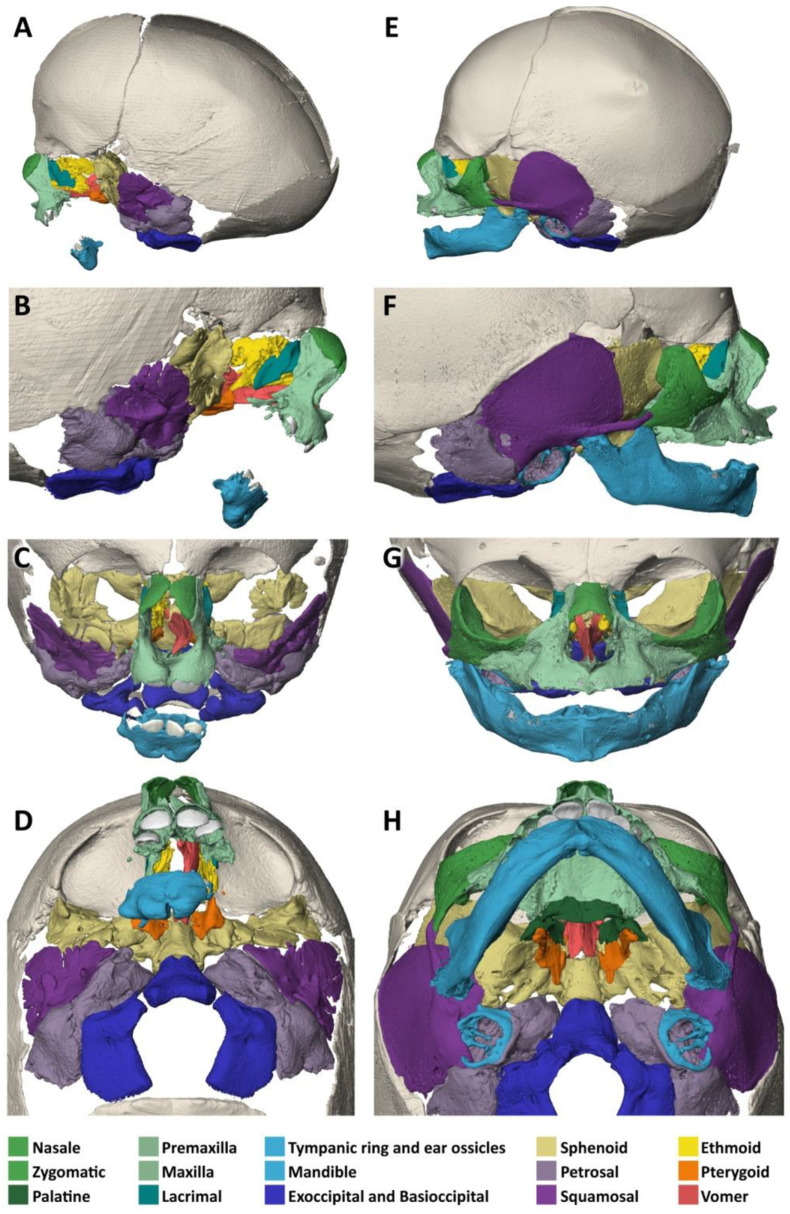
Specimen 2 and reference: Micro-CT-based 3D reconstruction of the skulls. Osseous defects in specimen 2, acrofacial dysostosis syndrome of Rodriguez (**A**–**D**). Reference 2, unaffected 7-gestational-month-old preterm; Terzer collection (**E**–**H**). Lateral view from the left (**A**,**E**) and from the right (**B**,**F**). Frontal view (**C**,**G**). Inferior view of the skull base (**D**,**H**). Individual cranial bones are color-coded to facilitate comparison. Note the absence of the zygomatic bones and the rudimentary lateral maxilla and mandible.

On micro-CT analysis (Figure 6A–H), there are normal calvarial bones in comparison to a 7-gestational-month-old normal fetus.

- The nasal bone (dark green) is not yet fused in the midline and has a caudal and rostral cleft. The vomer (red) is strongly curved to the left. The upper lamellae are not yet fused, the base is rounded, and a basal plate is missing in the absence of a secondary palate. The cartilaginous part of the nasal septum is also bent to the left and narrows the left nasal passage.
- The upper jaw (light green) is missing most of its lateral parts, including the osseous crypts of the canines and molars, orbital segments, infraorbital, incisive and palatine foramina and the zygomatic processes. Some rudimentary fragments of the lateral maxilla are attached to the lateral edges of the premaxilla, more pronounced on the left than on the right side. The alveolar ridge of the premaxilla contains four primary incisors within incompletely developed bony crypts. The nasal part shows no significant changes, but the primary palate including the incisive foramen is absent. The secondary palate including the palatal bones (dark green) is completely missing. There is also no soft palate. There is a direct connection between the oral cavity and the nasal conchae.
- The lower jaw (light blue) is completely missing its lateral parts, including the nerve canals and the mandibular and mental foramina. A small central part is incompletely fused in the lower and dorsal midline and, like the maxilla, has an underdeveloped alveolar ridge. The upper edge of the alveolar bone ends halfway up the teeth. There is one right and two left primary incisors. The second primary incisor on the right side is slightly distant from the first incisor and not enclosed in an ossified crypt, corresponding to the position of a canine. There are no molars.
- The *zygomatic bones* are completely absent. The greater wings of the *sphenoid bones* (beige) look severely dysplastic and fragmented. But the basal sphenoids, the presphenoids and orbitosphenoids appear relatively normal in shape. Fragmentation tends to affect more those parts of the sphenoid that are formed by intramembranous ossification. The squamosal parts of the temporal bones (deep violet) appear hypoplastic with wide open temporosphenoidal sutures and only rudimentary right and left zygomatic processes. The petrosal and tympanic part of the temporal bones (light violet) are present. The lacrimal bones (turquoise) are greatly enlarged or fused with the hypoplastic ethmoid bones (yellow). The orbital roofs, formed by the frontal bones, are of normal shape. The medial walls are formed only by the lacrimal bones and parts of the ethmoid bones, as the corresponding parts of the maxilla are missing. The orbital floor and lateral walls are completely absent. These are normally formed by the maxilla, zygomatic, and sphenoidal bones. Foramina rotunda and ovalia of the skull base are not identifiable.
- The ears are lacking the upper part of the auricles. The length of the auditory canals is 2.2 mm on the right and 6.5 mm on the left side. In the middle ears, behind membranous structures without identifiable tympanic rings, possibly representing the tympanic membrane, there are very small, non-air-filled tympanic cavities, each containing a deformed stapes but lacking the malleus and incus. Rudimentary Eustachian tubes branch off, but their further course cannot be determined with certainty. They have connections to the pharynx. The inner ears are lying in close proximity to the middle ears and look completely normal, with well-developed cochlea and labyrinth (Figure 4D). Foramina rotunda and ovalia are present.

For the 3-dimensional animation of the micro-CT reconstruction of the skull, please refer to Supplementary Video S2.

### 3.3. Specimen 3 (MN.9948)

**Figure 7:**
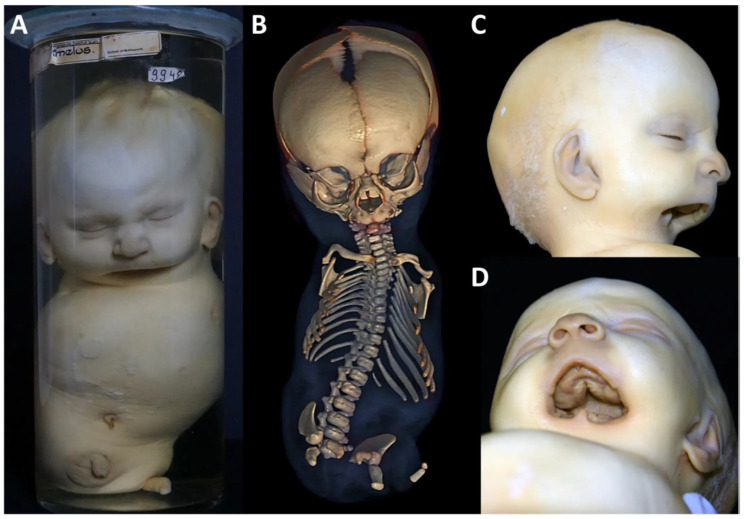
Specimen 3: Clinical diagnosis of tetra-amelia syndrome. Male newborn displaying tetra-amelia with a residual skin appendage on the left hip and severe bilateral transverse facial clefts (**A**). Whole-body CT with 3D reconstruction showing absent limb bones except for two small bone fragments within the limb appendage, hypoplastic scapulae and iliac bones, shortened, non-fused ischia and pubic bones, missing sacrum and coccyx and eleven ribs bilaterally (**B**). Transverse orofacial clefts and furrows towards the auditory canals (TC-7) and absence of the chin (**C**,**D**). (**A**) Reproduced from Boer et al. (2023) [12], with permission from Wiley, licensed by CC BY.

Clinical features: The male specimen with 27 cm CRL and 34.5 cm HC shows tetra-amelia with complete absence of the upper and lower limbs, except for a residual skin appendage of 4 cm length at the left lower hip (Figure 7A). Judging by the encrusted navel and dentition, the newborn is mature (~40 GW), though small for date, and must have lived postnatally for more than a week. It presents with a slightly enlarged skull in relation to the body length, bulging forehead and macrostomia due to a bilateral transverse oral cleft. On the left, the cleft merges midway into a fine linear cheek groove that fuses with the openings of the stenotic auditory canal. On the right, the groove is not visible (Figure 7A,C,D). The ears are slightly enlarged, low set with slightly hypoplastic upper helix on the right side but otherwise well-developed auricles at most according to grade 1 [25,26]. There is a complete cleft of the soft palate. There is also a small tongue and a missing chin and lower lip, with a residual left paramedian cone-shaped prominence at the lower mouth opening (Figure 7C,D). The neck is short and broad, the chest is prominent with hypoplastic nipples, the left nipple is displaced downward along the milk line, the abdomen is sunken, the testes are undescended and there is a shawl scrotum. A left-sided diaphragmatic defect is recognizable radiographically. X-rays and whole-body CT show missing limb bones except for two small bone fragments within the left lower limb appendage, hypoplastic scapulae and iliac bones, shortened and unfused ischiatic and pubic bones, and eleven ribs bilaterally (Figure 7B). We assigned the features described to tetra-amelia syndrome (OMIM #273395, # 618021) with orofacial cleft Tessier no. 7.

**Figure 8:**
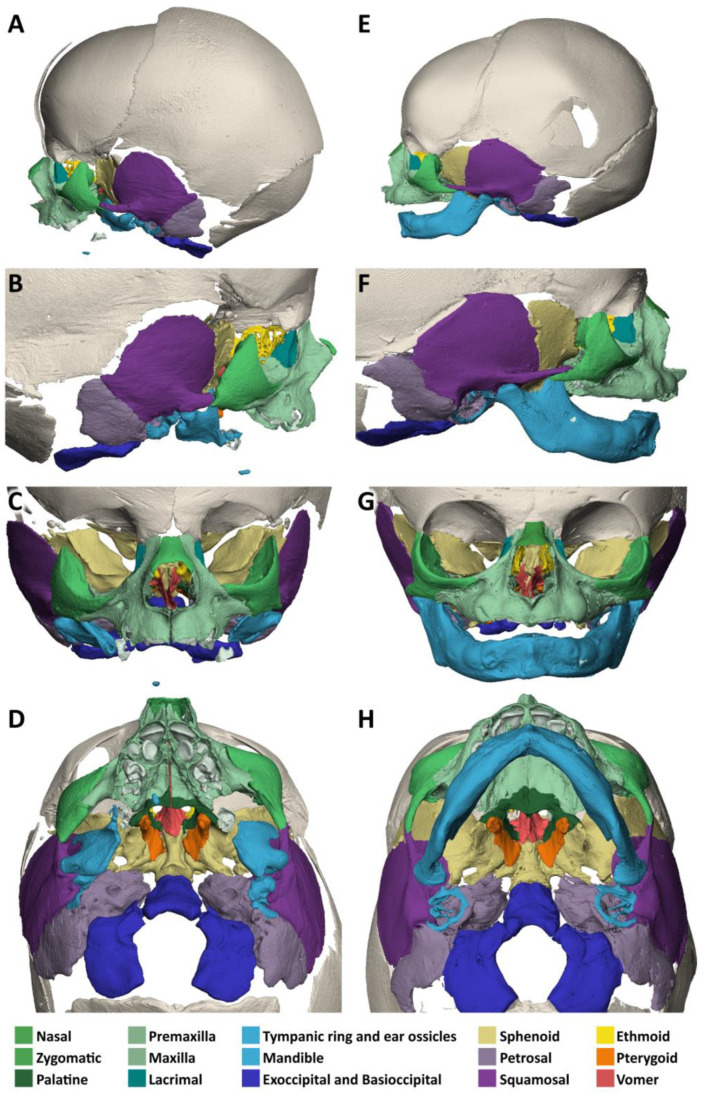
Specimen 3 and reference: Micro-CT-based 3D reconstruction of the skulls. Osseous defects in specimen 3, tetra-amelia syndrome (**A**–**D**). Reference individual 3, 1 month old; Terzer collection (**E**–**H**). Lateral view from the left (**A**,**E**) and from the right (**B**,**F**). Frontal view (**C**,**G**). Inferior view of the skull base (**D**,**H**). Individual skull bones are color-coded to facilitate comparison. Note the mildly hypoplastic, narrowed maxilla and the absence of most of the mandible with residual condylar processes.

Micro-CT analysis shows a normal-sized skull and nasal (green) bones, when compared to a normal neonate (Figure 8A–H).

The vomer (red) is approximately normal in shape, but the base is hypoplastic with only the right lamella contacting the palatal roof to the right of a narrow median palatal fissure. The nasal septum is of normal shape.

The upper jaw (light green) appears hypoplastic, and the palate is V-shaped and high and narrowed by at least a quarter in length and width. The palatine bones (dark green) are present. The median and transverse palatine sutures are widened. The deciduous incisors, canines and first and second molars are crowded in the narrow maxilla. The alveolar pockets are age-appropriate and the incisive, palatine and infraorbital foramina are visible.

The lower jaw (light blue) lacks the mandibular body and mental foramina. The mandibular rami and angles are rudimentary, with deformed coronoid and condylar processes. The mandibular foramina can be identified. The temporal joint sockets are visible. However, the temporomandibular joints appear to be immobile because the mandibles rest against the tympanic bones. There is no bony alveolar ridge. On both sides, a presumably second deciduous molar, not surrounded by a bony crypt, is visible without any connection to the mandible.

The zygomatic bones (middle green) show a shortening of the temporal processes and the temporal bones (deep violet) of the zygomatic processes, more so on the left than on the right. The tympanic and petrosal part of the temporal bones (light violet) are relatively well-developed, but the temporoparietal, temporozygomatic and frontozygomatic sutures are clearly widened. The sphenoid bones (beige) are slightly hypoplastic and of apparently normal shape. Foramina rotunda and ovalia are present. The orbits are elongated and distorted downward, with a distinct cleft between the zygomatic bone and the frontal bone which corresponds to a TC-8.

The ears are low set with well-developed auricles. The length of the stenotic auditory canals is 5 mm on the right and 3.5 mm on the left side. There are no demarcated eardrums in the presence of thickened tympanic rings that lack their typical ring structure. Slightly offside are the normal-sized, non-air-filled cavities of the middle ears. They each contain three ossicles. The non-air-filled Eustachian tubes show no connection to the pharynx. Both inner ears are well developed with normal cochleas and semicircular canals (Figure 4F). For a 3-dimensional animation of the micro-CT reconstruction of the skull, please refer to Supplementary Video S3.

## 4. Discussion

This comparative morphological study revealed significant differences in the severity of bone defects and dental maldevelopment associated with lateral facial clefts. In specimen 1 with Treacher Collins syndrome (TCS), the main bones affected are maxilla and the zygomatic bones; in specimen 2 with acrofacial dysostosis syndrome of Rodriguez (AFD-Rod), we found the most severe bone defects affecting the maxilla, the zygomatic bones and the mandible; and in specimen 3 with tetra-amelia syndrome (TETAMS), it is only the mandible that shows serious defects. In addition, other facial bones were shown to be affected by changes in size and shape, suture extension, fusions and disintegration. The differences in the type and combination of facial bone defects may be influenced by the different underlying gene defects and the role these genes play in facial development. Molecular studies in these specimens were not possible due to previous long-term fixation in formalin. Formalin has been demonstrated to impede DNA analysis through several mechanisms. These include DNA fragmentation, cross-linking between DNA and proteins, reaction with hydrogen bonds, denaturation of DNA proteins and methylation of nucleic acids [27]. So, we had to rely entirely on our clinical syndrome diagnoses. However, to better understand the processes responsible for the facial bone defects in our syndromic specimens, it is crucial to include the known genetic background of these disorders in our considerations. This may, of course, limit the informative value of this approach when applied to our syndromic, molecularly non-confirmed specimens.

Specimens 1 and 2 have in common a severe facial bone defect associated with Tessier cleft no. 6, 7 and 8. This condition may be seen in mandibulofacial (MFD) and acrofacial dysostosis (AFD), Goldenhar syndrome and hemifacial microsomia; the latter two are currently subsumed under *SF3B2* loss of function-associated craniofacial microsomia. Associated facial bone anomalies in specimen 1 and 2 include, in contrast to specimen 3, the absence of the lateral maxilla and zygomatic bones. The main difference between specimens 1 and 2 concerns other midfacial bones, including the sphenoid, ethmoid and temporal bones, and the mandible. In specimen 1, the midfacial bones are markedly underdeveloped and fused to a single mass and the mandible is present but shortened and contains all the mandibular primary teeth. In specimen 2, the sphenoid bones are fragmented, and the lateral parts of the mandible are missing, leaving a median segment with four primary incisors. The morphological similarities between MFD-TCS and AFD-Rod could be due to molecular genetic mechanisms that cause similar disturbances to cranial neural crest cells.

Autosomal dominant TCS results from haploinsufficiency of the genes *TCOF1* (78–93%), *POLR1D* and *POLR1B* (8%) (TCS 1, 2, 4). An autosomal recessive inheritance based on a biallelic loss of function of *POLR1C* (TCS 3) and a homozygous missense mutation in *POLR1D* have also been described [28,29,30,31]. TCS is a ribosomopathy. The *TCOF1* gene product, a Treacle protein, and subunits of RNA polymerases I and III (genes *POLR1D*, *POLR1B*, *POLR1C*) play an important role in ribosomal biogenesis and rRNA processing [28,29,32]. These proteins are particularly important for the survival of neural crest cells, and their depletion leads to oxidative stress-induced DNA damage and p53-associated apoptosis of these cells [29,33]. Accordingly, it has been demonstrated that genetic and pharmacological p53 inhibition, as well as antioxidant supplementation, precludes oxidative stress-induced cell death in the neuroepithelium. This significantly ameliorated, or even prevented, craniofacial anomalies in TCS mouse embryos [34,35,36]. Interestingly, dysfunction of the *POLR1A* gene, which encodes the largest RNA polymerase I subunit, results in another clinically overlapping ribosomopathy—autosomal dominant acrofacial dysostosis, Cincinnati type (AFD-Cin), which can include highly variable degrees of mandibulofacial dysostosis, limb defects, and abnormal neurodevelopment [37].

Autosomal dominant AFD-Rod is caused by haploinsufficiency of the gene *SF3B4* [38,39]. It was previously considered allelic to Nager syndrome (AFD1), with an intermediate *SF3B4* phenotype having also been reported [38,40]. However, more recent observations concerning the occurrence of AFD1 and AFD-Rod within the same family, caused by the same *SF3B4* mutation, suggest that these conditions are part of a spectrum of *SF3B4*-related disorders that can vary in severity, rather than separate allelic entities [6]. While the limb anomalies in AFD1 are mostly radial defects, cases of AFD-Rod show more severe limb deficiencies or even phocomelia. The craniofacial features and internal malformations in AFD-Rod are also more severe and may result in respiratory failure and perinatal death [6,41].

The *SF3B4* gene encodes the mRNA spliceosomal protein splicing factor 3B, subunit 4 [40]. Spliceosomes remove introns from pre-mRNA molecules. Limited SF3B4 functioning alters pre-mRNA splicing and thus reduces gene expression, especially during craniofacial and limb development [38]. Spliceosomal MFD also includes autosomal dominant mandibulofacial dysostosis with microcephaly and neurodevelopmental delay (MFDM). The responsible gene *EFTUD2* encodes a spliceosomal GTPase essential for neural crest development [42,43].

The ‘MFD spliceosomopathy genes’ are responsible for the formation and maintenance of neural crest progeny, in particular the cranial neural crest-derived cartilages [43]. Their knockdown, as shown in *Xenopus* embryos, broadly interferes with the translation of the corresponding mRNA and thus does not directly model the variant proteins but down-regulates the neural crest-specific genes [44]. Inactivation of *Eftud2* in the neural crest cells of the Wnt1-Cre2 mouse line resulted in brain and midface anomalies strongly resembling MFDM. This was associated with increased nuclear p53 enhanced expression of p53-activated genes and increased cell death, without affecting cell proliferation [45]. Knockdown of *EFTUD2* in a human cell line resulted in reduced proliferation, increased sensitivity to endoplasmic reticulum stress and enhanced apoptosis [43,46]. The same accounts for *SF3B4* mutations [43]. Notably, some of these genes (*Eftud2*, *Sf3B4*, *Snrpb*, *Txn14*) are also expressed in the developing cranial placodes, the precursors of sensory organs [44,47].

Both ribosomopathies and spliceosomopathies are associated with DNA damage signaling and repair mechanisms. In ribosomopathies, Treacle interacts with the MRN complex, which assembles at sites of double-strand DNA breaks. This limits oxidative stress-induced cell death. In spliceosomopathies, SF3B accumulates with proteins that regulate the splicing of pre-mRNA from genes involved in DNA repair [43].

Specimen 3 exhibits a unique combination of autosomal recessive tetra-amelia syndrome (TETAMS) with severe transverse facial cleft Tessier no. 7 and an almost complete absence of the mandible, of which only the very proximal parts are present (agnathia). To our knowledge, only two short case reports have been published with the same unusual combination of defects—a female fetus at 28 weeks gestation showing severe tetradysmelia in association with otocephaly, holoprosencephaly and lung hypoplasia, and a male fetus at 18 weeks with tetra-amelia, agnathia and left diaphragmatic hernia [48,49]. Two siblings with tetra-amelia from consanguineous parents and a third affected patient have been described as having a ‘big mouth’ [50,51]. Moreover, many patients with tetra-amelia syndrome have a significant microretrognathia, lack of medial mandibular fusion and some also have a common cleft lip and palate, ankyloglossia, choanal atresia, lacrimal duct malformation and absent nipple [7]. This suggests some common developmental mechanisms for the growth of the limb buds and facial prominences. A few patients with tetra-amelia syndrome were reported to have survived the neonatal period, some even into adulthood [52,53]. In specimen 3, in addition to TC-7, agnathia and tetra-amelia, there is an incomplete cleft of the posterior palate and displaced hypoplastic nipples. Other internal organs could not be assessed.

To date, biallelic loss-of-function mutations in two genes have been reported to be causally associated with human tetra-amelia syndrome, *WNT3* [54] and *RSPO2* [55]. A homozygous 50-kb deletion disrupting the *rspo2* gene has also been identified as the cause of tetradysmelia (severe reduction defects of all limbs) in a backcrossed family of Holstein Friesian cattle. Some affected calves had additional jaw abnormalities such as a lack of mandibular midline fusion or an underbite due to brachygnathia superior [56]. Indeed, human patients and animal models with *RSPO2/rspo2* gene mutations have shown that the clinical spectrum of tetra-amelia syndrome is broad and that hypomorphic mutations, such as some missense mutations, result in tetradysmelia and milder facial anomalies [55,56].

The *RSPO2* gene is highly expressed in limb buds, but also in branchial arches and the lung mesenchyme, and it plays a fundamental role in craniofacial, limb and lung development in humans [55]. Suppressing the expression of its product R-spondin-2 promotes chondrocyte differentiation by increasing TGF-ß induction. R-spondin-2 is required for the activation and stabilization of the *Wnt/beta-catenin* pathway, either directly through LGR4-6 receptors or by inactivating RNF43 and ZNRF3, which mediate degradation of the *WNT* receptor. *WNT* is a large family of genes that encode secreted signaling proteins which are important for the regulation of cell growth, migration, differentiation and fate in embryonic development and oncogenesis. The *Wnt* signaling pathway is substantial for the formation of the apical ectodermal ridge and thus for the initiation and maintenance of proximal–distal limb outgrowth (*Wnt3a*), as well as for early facial development (*Wnt5a*). The highest expression of *WNT5a* is found in the lower facial prominences [15].

As with the facial bones, ear development was also differentially affected in our three syndromic specimens. The ear components develop from multiple progenitor structures. The middle and outer ears form from the ectodermal mesenchymally transformed neural crest cells of the first and second pharyngeal arches and the epithelial cells of the endodermal pharyngeal pouches and ectodermal pharyngeal clefts [19,20]. The development of the inner ears is initiated by the ectodermal otic placodes, well separated from the pharyngeal arches. Thus, the inner ears are not of neural crest origin and appear to develop independently. Anomalies, such as the clumped cochleae and the absence of three semicircular canals, observed only in specimen 1, are more likely to present a separate feature.

It should be noted that two of the three middle ear ossicles, the malleus and the incus, are mainly formed from the dorsolateral part of Meckel’s cartilage (MC). MC arises from the mesenchymal neural crest cells of the ventral, mandibular part of the first pharyngeal arch at the level of the first molar tooth germ. It extends as a continuous rod-shaped mandibular template ventromedially and dorsolaterally throughout the lower jaw. The distal ends fuse in the midline to form the symphysis of the mandible, the proximal ends develop into the primordia of the malleus and incus, and the intermediate zones regress or degrade later into fibrous tissue [57]. The structures arising from the ends of the MC undergo endochondral ossification. In contrast, almost the entire body of the mandible undergoes intramembranous ossification, induced and guided by the MC. Therefore, it is likely that proximal truncation of the MC due to impaired dorsolateral growth or prolonged degradation is responsible for the absence of the malleus and incus and the defective ossification of the posterior and possibly intermediate mandible, as in specimens 1 and 2. The same defective processes, when affecting the distal ends of the MC, result in micrognathia, dysgnathia or even agnathia with the presence of middle ear ossicles and rudimentary ascending mandibles, as in our specimen 3 [20,57]. Abnormalities of the mesenchymal structures of the auricles, auditory canals, eardrums and tympanic rings may be associated with defects of the MC-derived mandible and ossicles. Although their development is independent and not directly related to MC, their anomalies arise from the same mesenchymal neural crest cell disorders due to their common pharyngeal arch origin. The lack of major ear defects in agnathia, as observed in our specimen 3, is comparable to the agnathia-otocephaly complex (AGOTC), in which similar mild ear abnormalities have been described, except for auricular malposition or even midline fusion, and postnatal audiograms showed mostly normal hearing [58]. AGOTC is characterized by additional severe microstomia, micro- or aglossia, and even holoprosencephalia. The condition is caused by mutations in the *PRRX1* and *OTX2* genes [59].

An interesting finding is the presence of several developing teeth in the absence of a surrounding bony crypt. Under physiological conditions, tooth development is closely linked in time and space to the formation of the alveolar bone of the developing jaw: Shortly after the fusion of the facial processes, the first signs of ossification can be seen in the mandible and maxilla. Tooth germs generally do not begin to mineralize around week 13 [60]. In specimen 1, a right maxillary first molar at the level of the absent orbital floor is located in the soft tissue close to the lateral incisor, which is surrounded only by a rudimentary bony crypt. Schlump et al. [61] also reported some dental germs in the orbital floor of a patient with diagnosed Treacher Collins syndrome. These tooth germs, however, seemed to be encased by an osseous crypt. Specimen 2 shows a right mandibular second incisor (or canine) located above the rudimentary mandibular crypt, slightly distant from the right first incisor. In specimen 3, we found two developing mandibular molars close to the rudimentary, deformed mandibular rami, surrounded only by soft tissue. These findings suggest that tooth development, and in particular tooth germ mineralization, is not restricted to the same regulatory mechanism as jaw bone formation.

To the best of our knowledge, this is the first micro-CT imaging study on lateral facial clefts. Its application to museum specimens allows for the underlying skeletal structures to be analysed in great detail. This level of accuracy cannot be reached by other radiological techniques. Therefore, it provides novel insights into the true nature of these severe facial malformations and the co-occurrence of ear anomalies and disturbances in tooth development, which have not been reported in the literature before. The significance of this study lies in its dual approach, combining high-resolution imaging for anatomic–morphological analysis with genetic and developmental perspectives.

However, our study has several limitations that should be addressed. The analysis was restricted to only three exemplary specimens. Focussing on the skeletal involvement of lateral facial clefts, associated soft tissue defects have not been considered. This remains an issue that will be addressed in future studies. A corresponding pilot study is already in progress. The micro-CT technique presented cannot be used on living patients because of the excessive radiation exposure. It was not possible to conduct DNA analyses to confirm the clinical syndrome diagnosis in our formalin-fixed museum specimens. This is a considerable disadvantage when studying museum specimens, as it limits analyses to descriptive morphological observations. In addition, the severe forms of these syndromes presented are extremely rare, which is why there is a lack of clinical and surgical experience in such cases.

## 5. Conclusions

Lateral facial clefts of Tessier no. 6 to 8, as observed in our three syndromically affected museum specimens, showed severe underlying bone defects on micro-CT, allowing us to determine the morphological extent of the developmental disturbances. TCS and AFD-Rod belong to a remarkable group of ribosomopathies and spliceosomopathies. In these conditions, facial bone defects occur as a result of disruptive events due to oxidative stress-induced DNA damage and apoptosis of cranial neural crest-derived cells. This led to a more generalized and irregular pattern of bone defects in specimen 1 and 2, and particularly affected the maxilla with absence of the zygomatic bones, atypical shape, fusions or furrowing of other midfacial bones, abnormal ears, and, to a lesser extent the mandibles. On the other hand, mutations in the genes responsible for TETAMS cause a more distinct pattern of disturbances in the regulation of neural crest cell proliferation, migration and differentiation in limb buds and craniofacial lower prominences. As shown in specimen 3, these disturbances particularly affected the Meckel’s cartilage-dependent mandible and largely spared the bones deriving from the maxillary processes, and the ears.

Thanks to the advances in prenatal diagnosis, full-term presentations of severe malformations such as those described in this study are becoming increasingly rare. Consequently, clinicians mainly deal with individuals who are less severely affected and viable. Craniofacial anomalies are often genetic and exhibit variable expressivity, resulting in a broad spectrum of clinical presentations. However, to better understand the underlying developmental processes and pathogenic mechanisms, it is essential also to consider the most severely affected non-viable individuals. Our findings provide detailed 3D mapping of severe phenotypic manifestations of lateral facial clefts, offering crucial insights into the broad spectrum of craniofacial disruptions. To fully understand the morphological implications of these conditions, specimen collections like those of the Narrenturm are an invaluable source of knowledge, especially when studied with state-of-the-art imaging techniques.

## Figures and Tables

**Table 1:** The skeletal features observed in our three specimens compared with those of Treacher Collins syndrome (TCS), acrofacial dysostosis syndrome of Rodriguez (AFD-Rod), and tetra-amelia syndrome (TEAMS).

Skeletal Features	Specimen 1	TCS	Specimen 2	AFD-Rod	Specimen 3	TETAMS
CLEFT TYPE	**TC-6, 7 and 8**	TC-6, 7, 8 (X-ray) (eyelid coloboma and macrostomia)	**TC-6, 7 and 8**	TC-6 ? (defect of lower eyelashes)	**TC-7**	TC-7 (1 patient)
MAXILLA						
Lateral maxilla	**A**	hypoplastic	**rudimentary**	hypoplastic	**NN**	N
Premaxilla	narrow, severely hypopl., displaced upwards	midline hypoplasia	narrow, hypopl.	short philtrum	narrow, hypopl.	n.d.
Primary palate	hypoplastic	n.d.	**hypoplastic**	n.d.	hypoplastic	n.d.
Secondary palate	**A**	cleft, high arched	**A**	cleft, high arched	narrow, cleft, high arched,	cleft lip and palate
Tongue	N	glossoptosis	glossoptosis	n.d.	hypoplastic	Ankyloglossia
Dentition	defective	defective	defective	n.d.	**N** crowded	n.d.
Choanae	stenosis	atresia/stenosis	atresia	stenosis/atresia	**N**	Atresia
MANDIBLE						
Corpus	shortened, sev. micrognathia	hypoplastic, sev. micrognathia	defective, sev. micrognathia	hypoplastic, sev. micrognathia, microstomia	**A**	micrognathia, agnathia in 2 cases, no medial fusion
Rami/angles	hypoplastic	hypoplastic	**A**	n.d.	**rudimentary**	n.d.
Condylus	deformed	n.d.	**A**	n.d.	**deformed**	n.d.
Coronoid proc.	**hypoplastic**	n.d.	**A**	n.d.	**deformed**	n.d.
Dentition	**N**	defective	**defective**	n.d.	**A**	n.d.
OTHER CRANIAL BONES						
Zygomatic bone	**A**	A/ hypoplastic	**A**	malar hypoplasia	**N**	**NN**
Zygomatic arch	**A**	hypoplastic	**A**	hypoplastic	**shortened**	NN
Sphenoid bone	hypoplastic/ fused	hypoplastic	hypoplastic/ fragmented	hypoplastic	N	NN
Nasal bone	rudimentary	hypoplastic	caudal and rostral cleft	prominent nose	N	NN
Ethmoidal bone	hypoplastic/ fused	hypoplastic	hypoplastic/ fragmented	hypoplastic	N	lacr. duct abnomal
Temporal bone	**hypoplastic/** **fused**	hypoplastic	**hypoplastic/** **fragmented**	hypoplastic	NN	NN
Orbits	A (lower rim)	hypoplastic (upper rim)	A (lateral and lower rim)	hypoplastic (upper rim)	elongated and distorted downward	hypertelorism and deep-set eyes
EAR STRUCTURES						
Inner ear	**malformed cochlea** **and labyrinth**	n.d.	N	n.d.	N	N
Middle ear	**rudimentary**	conductive, hearing loss	**rudimentary**	n.d.	N	N
Ossicles	**A**	n.d.	**A** **(incus and malleolus)**	n.d.	N	N
Eustachian tube	N air-filled	n.d.	**rudimentary,** **non-air-filled**	n.d.	N non-air-filled	n.d.
Tympanic membrane	**A**	n.d.	**A**	n.d.	not identifiable	N
Auditory canal	blind fistulas	blind fistulas	stenotic	blind fistulas	stenotic	N
Auricles	**A** **ear tag**	hypo-/dysplastic, and ear tags	**absent upper auricles and 3-lobed lower tags**	low set and hypo-/dysplastic	N, low set and	low set, mildly hypoplastic
	**MO grade IV**	MO grade I–III	**MO grade III**	MO grade I–III	**macrotia**	MO grade I
EXTRAFACIAL SKELETON	N	N	**tetraphocomelia absent pelvis and hypopl.scapulae**	phocomelia, hypoplast. pelvis and scapulae	**tetra-amelia,** hypoplastic pelvis	tetra-amelia/ tetradysmelia, hypoplastic pelvis

A = absent; AFD-Rod. = Acrofacial dysostosis type Rodriguez; hypo-dyspl. = hypoplastic/dysplastic; MO = microtia; N = normal; n.d. = not yet described; NN = nearly normal; sev = severe; TC = Tessier cleft; TCS = Treacher Collins syndrome; TETAMS = tetra-amelia syndrome. The sources of normative data standards are taken from Allanson et al., 2009 [25] and Hall et al., 2007 [26].

## Data Availability

Data that support the findings of this study are included in this article. Further enquiries can be directed to the corresponding author.

## Supplementary Materials

The following supporting information can be downloaded at https://www.mdpi.com/article/10.3390/biology14070872/s1. Table S1: Morphometric parameters measured in the three specimens analyzed and the respective reference individuals. Video S1: Animated 3D reconstruction of the micro-CT scan of Specimen 1 (Clinical diagnosis of Treacher Collins syndrome). Video S2: Animated 3D reconstruction of the micro-CT scan of Specimen 2 (Clinical diagnosis of acrofacial dysostosis syndrome of Rodriguez). Video S3: Animated 3D reconstruction of the micro-CT scan of Specimen 3 (Clinical diagnosis of tetra-amelia syndrome).

## Abbreviations

The following abbreviations are used in this manuscript:

AFD1	Nager syndrome
AFD-Rod	Acrofacial dysostosis of Rodriguez
AGOTC	Agnathia otocephaly complex
CHL/CRL/HC	Crown-heel length/crown-rump length/head circumference
DW/GW	Developmental week/gestational week
EFTUD2	Elongation factor Tu GTP-binding domain-containing 2
LGR	Leucine-rich repeat-containing G protein-coupled receptor
MC	Meckel’s cartilage
MFD	Mandibulofacial dysostosis
MFDM	Mandibulofacial dysostosis with microcephaly
MRN complex	Accumulation of Mre11, Rad50 and Nbs1 at sites of double-strand DNA breaks
OTX	Orthodenticle homeobox X1
POLR	Polymerase
PRRX	Paired-related homeobox
RNF	Ring finger protein
RSPO	R-Spondin
SNAI	Snail family transcriptional repressor
SOX	SRY-BOX
SF3B4	Splicing factor3 subunit 4
TCOF1	Treacle ribosome biogenesis factor
TC	Tessier cleft
TCS	Treacher Collins syndrome
TETAMS	Tetra-amelia syndrome
tfap2e	Transcription factor AP2-epsilon
TGF	Transforming growth factor
WNT	Wingless-related integration site
ZNRF’	Zinc finger and ring finger protein
