# Supplementary material for: Facial Bone Defects Associated with Lateral Facial Clefts Tessier Type 6, 7 and 8 in Syndromic Neurocristopathies: A Detailed Micro-CT Analysis on Historical Museum Specimens
Source: Biology (Basel). 2025 Jul 17;14(7):872. doi: 10.3390/biology14070872 (PMC12292342; doi:10.3390/biology14070872)
Supplement: Supplementary file 1 [file biology-14-00872-s001.zip › biology-3698693-supplementary/Supplemental Table S1.pdf]

Supplemental Table S1

Anatomical landmarks were manually set in the 3D reconstructions of the μCT scans, and the distances between them were automatically calculated. The following morphometric parameters were assessed: (1) upper facial height, defined as the straight-line distance between the Nasion and Prosthion, (2) facial height including the mandible (between the Nasion to Gnathion), (3) length of the hard palate (between the Orale to the Staphylion), 4) width of the hard palate (between the most medio-distal point of the tuber maxillaris) and (4) length of the mandible (defined as the distance between the most anterior extension of the mandible to the intersection of a virtual line drawn between the Gonion, i.e. angulus mandibulae, in the midsagittal plane [1]. In some cases, the anatomical landmarks had to be adjusted according to the specific anatomical conditions of the specimens. Given that the age of the specimens and that of the reference individuals are not identical, the measurements, only allow for an approximate comparison without asserting general validity. The morphometric measurements are presented in **Supplemental Table S1**.

Supplemental Table S1: Morphometric parameters measured in the three specimens analysed and the respective reference individuals

Individual	**Estimated age at death** according to London Atlas & clinical indices (CRL, CHL, HC)	Upper facial height	Facial height including mandible	Length of hard palate	Width of hard palate	Length of mandible
**Specimen 1** Treacher Collins syndrome	40 weeks gestation (term neonate)	16.8	27.08	5.29 * (only premaxilla present)	5.78 *	20.32
**Reference Individual 1**	4 postnatal days	27.08	39.1	22.37	20.18	29.1
**Specimen 2** Acrofacial dysostosis syndrome of Rodriguez	30 weeks gestation (preterm)	21.80	30.7	1.76 * (only premaxilla present)	7.56 *	4.93 * (no angulus mandibulae present)
**Reference Individual 2**	28 weeks gestation (preterm)	20.83	30.28	15.41	15.96	26.95
**Specimen 3** Tetraamelia syndrome	1 postnatal week (term neonate)	26.05	not measurable (no mental region present)	16.91	12.08	13.90 *
**Reference Individual 3**	4 postnatal weeks	26.85	37.27	19.55	17.49	27.07

Abbreviations: GW: gestation week; CRL: crown-rump length; CHL: crown-heel length; HC: head circumference
*Anatomical landmarks were adjusted to the respective anatomical conditions of the specimens.
Please note that the age of the specimens and that of the reference individuals is not identical. The measurements, thus, only allow for an approximate comparison without asserting general validity.
The sources of the standards osteometric parameters are taken from Bräuner, 1988 [1].

References

[1] G. Bräuer (1988) 2. Osteometrie. a) Kraniometrie; in: *Anthropologie. Handbuch der vergleichenden Biologie des Menschen Band 1*, R. Knußmann (Editor), Gustav Fischer Verlag, pp. 160–192.
